# Seasonal variation in cardiovascular and cerebrovascular hospitalizations across climatic zones in Northern and Southern Israel

**DOI:** 10.1186/s12872-026-05705-z

**Published:** 2026-03-24

**Authors:** Shira Deich, Michael Friger, Nathalia Bilenko, Yaakov Henkin, Doron Aronson, Gregory Telman, Sergey Yalonetsky

**Affiliations:** 1 Department of Epidemiology, Biostatistics and Community Health, Faculty of Health Sciences, Ben-Gurion University of the Negev, Beer-Sheva, Israel; 2 Department of Cardiology, Soroka University Medical Center, Ben-Gurion University of the Negev, Beer-Sheva, Israel; 3 The Ruth and Bruce Rappaport Faculty of Medicine, Technion, Israel Institute of Technology, Haifa, Israel; 4 Department of Cardiology, Rambam Healthcare Campus, Academic Hospital, Haifa, Israel

**Keywords:** Seasonality, Myocardial infarction, Stroke, Climate, Hospitalization, Israel, Trend-harmonic analysis

## Abstract

**Background:**

Seasonal variation in cardiovascular and cerebrovascular diseases (CCD) is well documented, with higher morbidity in winter. Israel’s contrasting climates—Mediterranean in the north and semi-arid in the south—offer a unique setting to examine associations between seasonality and hospitalizations.

**Methods:**

We conducted a 19-year retrospective ecological study using daily hospitalization data from Rambam Medical Center (RMC, north) and Soroka Medical Center (SMC, south) between 2000 and 2018. Hospitalizations were classified as acute myocardial infarction (AMI; ICD-9 410.0–410.9), ischemic stroke (ICD-9 434.90–434.91), or hemorrhagic stroke (ICD-9 430–431). Trend-Harmonic (Cosinor) analysis with Poisson or Negative Binomial regression models was applied to assess seasonal and weekly patterns.

**Results:**

A total of 88,101 hospitalizations were analyzed (RMC: 42,971; SMC: 45,130). AMI admissions showed significant winter peaks at both centers (RMC IRR = 1.107, *p* < 0.001; SMC IRR = 1.145, *p* = 0.002) and summer declines (RMC IRR = 0.946, *p* = 0.010). Ischemic stroke showed a modest winter increase in the north (IRR = 1.046, *p* = 0.023), while hemorrhagic stroke showed no significant seasonal variation. Daily mean AMI hospitalizations were highest in winter (3.2 at RMC; 5.1 at SMC) and lowest in summer (2.7 at RMC; 4.0 at SMC).

**Conclusions:**

AMI hospitalizations in Israel consistently peak in winter across diverse climates, highlighting robust seasonal associations in cardiovascular morbidity. These findings support targeted preventive strategies, including public health interventions and hospital preparedness, during colder months.

## Introduction

Cardiovascular and cerebrovascular diseases (CCD), including acute myocardial infarction (AMI) and stroke, remain major causes of morbidity and mortality worldwide [1]. Despite advances in prevention and treatment, these diseases continue to place a heavy burden on public health systems. In Israel, heart disease is the second leading cause of death after cancer, accounting for about 15% of all deaths. Each year, around 25,000 hospitalizations occur due to myocardial infarction, and in 2020, there were 18,373 cases of acute stroke, including ischemic stroke, transient ischemic attack (TIA), and cerebral hemorrhage [2–5].

Seasonal variation in cardiovascular events is well documented. Many studies have shown that hospitalizations for AMI and stroke peak during winter and decline in summer. Similar findings have been reported worldwide, including in Europe, North America, and Asia. For example, Vallabhajosyula et al. observed a clear seasonal pattern in AMI hospitalizations over 18 years of U.S. data, with higher rates and worse outcomes in winter. Proposed mechanisms include physiological responses to cold, such as increased blood pressure, higher blood viscosity, and inflammatory or hormonal changes. Conversely, exposure to high temperatures may also raise cardiovascular risk in warmer climates, indicating that both cold and heat can increase cardiovascular risk [6].

Israel provides a unique opportunity to study these seasonal effects due to its diverse climatic zones within a small area. The northern and central regions have a Mediterranean climate with mild, wet winters and hot, dry summers, while the southern region, especially the Negev Desert, features a semi-arid climate with more extreme temperature fluctuations and greater day–night variability [7]. These conditions enable researchers to observe how seasonal patterns in cardiovascular and cerebrovascular hospitalizations vary across different climatic environments.

Previous Israeli studies have demonstrated seasonal trends in certain diseases or regions. Telman et al. found increased rates of ischemic stroke and in-hospital mortality during winter in northern Israel [8]. Libruder et al. identified a winter peak in the incidence of ischemic stroke, TIA, and intracerebral hemorrhage among older adults [9]. In the semi-arid south, Yackerson et al. reported that AMI admissions were highest in winter and lowest in summer [10]. However, these studies were limited in scope, focusing either on a single condition or a specific area, and did not offer a comprehensive view of seasonal hospitalization patterns across various climatic regions in Israel.

To address this gap, the present study aims to describe and analyze seasonal and periodic patterns of hospitalizations due to acute myocardial infarction and both ischemic and hemorrhagic strokes in two distinct regions of Israel—the northern (Mediterranean) and southern (semi-arid) zones. Rather than directly comparing the two areas, the study focuses on identifying the presence, timing, and extent of seasonal variation in each region separately.

This study extends previous Israeli research by examining long-term seasonal patterns of cardiovascular and cerebrovascular hospitalizations across Israel’s major climatic regions using multi-year hospitalization data.

## Methods

### Study design and setting

This retrospective, population-based ecological study analyzed daily hospitalizations for acute cardiovascular and cerebrovascular diseases from January 1, 2000, to December 31, 2018, at two tertiary medical centers in Israel. Rambam Medical Center (RMC) is a 1,000-bed academic hospital serving over 2 million residents in northern Israel. It is located in a Mediterranean climate with mild, wet winters and hot, dry summers. Soroka Medical Center (SMC) is a 1,000-bed tertiary hospital serving approximately one million residents in southern Israel, situated in a semi-arid desert region with more extreme temperature fluctuations. These two hospitals provide representative coverage of Israel’s main climatic zones.

### Study population

We included all adult patients (≥ 18 years) admitted with a primary diagnosis of:

- Acute myocardial infarction (AMI): ICD-9 codes 410.0–410.9.
- Ischemic stroke: ICD-9 codes 434.90, 434.91.
- Hemorrhagic stroke: ICD-9 codes 430, 431.

Patients with incomplete admission dates or missing key demographic information were excluded.

### Data collection

Demographic and clinical data were extracted from electronic hospital records, including age, sex, admission date, primary diagnosis, and discharge outcome. Only the first admission per patient for each diagnosis was included to prevent double-counting.

### Definition of seasons

Seasons are defined according to Alpert et al. as follows: winter (December 7 to March 30), summer (May 31 to September 22), both lasting approximately four months (three months and 23 days); autumn (September 23 to December 6); and spring (March 31 to May 30), each lasting only two months (75 days and 61 days, respectively) [11]. All analyses were based on mean daily hospitalizations, thereby minimizing bias from unequal seasonal lengths.

### Outcome definition

The primary outcomes were the daily number of hospitalizations for each diagnosis (AMI, ischemic stroke, and hemorrhagic stroke) at each medical center, aggregated into time-series datasets representing daily hospital activity for each disease group and hospital. For graphical presentation, mean daily counts were calculated for each season.

### Exposure and temporal variables

The primary exposure of interest was time, captured by:

- Trend terms (linear and polynomial) to account for long-term changes;
- Harmonic terms (sine and cosine functions) to model annual and weekly cycles;
- Seasonal dummy variables for the four calendar seasons, with Fall as the reference category.

### Statistical analysis

Daily hospitalization counts were modeled using Tren-Harmonic and Cosinor regression analysis, which enable decomposition of time-series data into trend and periodic components [12].

Incidence Rate Ratios (IRR) were estimated from Poisson or Negative Binomial regression models, depending on the presence of overdispersion. In this time-series framework, IRRs represent the relative change in daily hospitalizations associated with a given temporal or seasonal component, compared with the baseline defined within the model itself, rather than a separate control period. This approach allows assessment of long-term trends and seasonal variation in daily admissions over the 18-year study period.

Goodness-of-fit was assessed using likelihood-based criteria (AIC, BIC).

To describe the long-term trend and periodicity of daily hospitalizations, we fitted the following Tren-Harmonic model:

$$\begin{aligned}&Y_{t}=a_{0}+a_{1}t+a_{2}t^{2}+a_{3}t^{3}+b_{1}\\&\cdot\cos\:(t\cdot\omega_{A})+b_{2}\cdot\sin\:(t\cdot\omega_{A})+c_{1}\\&\cdot\cos\:(t\cdot\omega_{w})+c_{2}\cdot\sin\:(t\cdot\omega_{w})+d_{1}S_{1}\\&+d_{2}S_{2}+d_{3}S_{3}+d_{4}S_{4}+\epsilon_{t}\end{aligned}$$

$a_{0}+a_{1}+a_{2}t^{2}+a_{3}t^{3}$ -  denotes the trend component, with *t* measured in days, and *a*_*0*_, *a*_*1*_, *a*_*2*_, *a*_*3*_ are coefficients.

$b_{1}\cdot\cos\:(t\cdot\omega_{A})+b_{2}\cdot\sin\:(t\cdot\omega_{A})$ - represents the annual harmonic cycle, capturing periodic variation within each year.

$c_{2}\cdot\cos\:(t\cdot\omega_{w})+c_{2}\cdot\sin\:(t\cdot\omega_{w})$ - represents the weekly harmonic cycle.

$d_{1}S_{1}+d_{2}S_{2}+d_{3}S_{3}+d_{4}S_{4}$ - represents a set of seasonal dummy variables, corresponding to the four calendar seasons.

$\epsilon_{t}$ denotes random error term (‘white noise’), assumed to be independent and normally distributed.

$\omega$= 2π/P, where P is the period of the cycle (e.g., a yearly cycle of 365 days and a weekly cycle of 7 days).

We utilize pairs of sine and cosine functions to represent different cycles, illustrating the periodic components of the time series. The trend component describes long-term changes over time, while harmonic terms quantify cyclical fluctuations. The residual ‘white noise’ refers to a random component that represents modeling errors.

Analyses were performed separately for RMC and SMC using SPSS software (version 29).

## Results

The demographic and clinical characteristics of the study population are presented in Table 1.

**Table 1:** Comparison of hospitalization characteristics among the study population at Rambam and Soroka Medical Centers (2000–2018)

	RMC	SMC	*P*
Total number of CCD hospitalizations	*N* = 42,971	*N* = 45,130	
Male	25,790 (59.9%)	29,061 (64.3%)	< 0.001
Female	17,172 (39.9%)	16,069 (35.5%)	< 0.001
Age (mean ± *SD*)	69.6 ± 13.89	67.96 ± 13.95	< 0.001
Myocardial infarction N (% of total)	18,229 (42.42%)	30,208 (66.93%)	< 0.001
Male	12,258 (67.2%)	20,822 (68.9%)	0.001
Female	5971 (32.8%)	9386 (31.1%)	< 0.001
Age (mean ± *SD*)	68.7 ± 13.85	67.08 ± 13.89	< 0.001
Ischemic stroke N (% of total)	21,440 (49.89%)	11,186 (24.79%)	< 0.001
Male	11,697 (54.6%)	6059 (54.2%)	0.607
Female	9743 (45.4%)	5108 (45.7%)	0.136
Age (mean ± *SD*)	71.02 ± 13.05	70.7 ± 13.24	0.037
Hemorrhagic stroke N (% of total)	3302 (7.68%)	3736 (8.28%)	0.001
Male	1835 (55.6%)	2169 (58.1%)	0.035
Female	1467 (44.4%)	1567 (41.9%)	0.035
Age (mean ± *SD*)	67.95 ± 13.95	66.5 ± 15.30	< 0.001

Continuous variables are presented with the mean and standard deviation (SD). Discrete variables are presented with counts and percentages. P-values were calculated using the Chi-square test for discrete variables and two-sample T-tests for continuous variables. *p* < 0.05

Between 2000 and 2018, a total of 42,971 CCD hospitalizations were recorded at RMC, and 45,130 at SMC.

Overall, males accounted for 59.9% of CCD hospitalizations at RMC and 64.3% at SMC (*p* < 0.001). The mean age of hospitalized patients was slightly higher at RMC (69.6 ± 13.9 years) compared to SMC (68.0 ± 14.0 years; *p* < 0.001).

Hospitalizations due to acute myocardial infarction (AMI) were more frequent at SMC (66.9% of all CCD admissions) than at RMC (42.4%, *p* < 0.001).

Conversely, ischemic stroke accounted for a higher proportion of hospitalizations at RMC (49.9%) than at SMC (24.8%, *p* < 0.001).

Hemorrhagic stroke represented a smaller proportion of admissions at both centers (RMC: 7.7%, SMC: 8.3%, *p* = 0.001).

At both hospitals, men were more frequently hospitalized for AMI (RMC: 67.2%; SMC: 68.9%), while sex distribution for ischemic stroke was relatively balanced.

The mean age of patients with ischemic stroke was higher than that of those with AMI or hemorrhagic stroke in both regions.

Table 2 presents the results of the trend-harmonic analysis for daily hospitalizations due to AMI, ischemic stroke, and hemorrhagic stroke at RMC between 2000 and 2018.

**Table 2:** Trend-harmonic analysis of daily CCD hospitalizations at RMC (2000–2018), by diagnosis

Variables	Acute Myocardial Infarction		Ischemic Stroke		Hemorrhagic Stroke	
	IRR^1^	*P*	IRR^1^	*P*	IRR^1^	*P*
Trend						
Intercept	1.760	< 0.001	2.561	< 0.001	1.221	0.013
* t*	1.000	< 0.001	1.000	0.007	1.000	0.818
* t* ^*2*^	1.000	< 0.001	1.000	0.808	1.000	0.924
* t* ^*3*^	1.000	0.013	1.000	0.103	1.000	0.874
Annual cycle						
Sin_year	1.021	0.052	0.985	0.124	1.002	0.923
Cos_year	1.009	0.412	0.994	0.512	1.027	0.275
Weekly cycle						
Sin_week	0.998	0.838	0.991	0.361	1.002	0.935
Cos_week	1.005	0.654	0.978	0.021	0.996	0.875
Seasonal cycle						
Winter	1.107	< 0.001	1.046	0.023	1.036	0.468
Spring	1.000	0.998	1.029	0.212	0.965	0.545
Summer	0.946	0.010	1.003	0.633	0.966	0.481
Fall (ref.)	1		1		1	

^1^Analysis was performed by Poisson regression

*P* < 0.05

A significant long-term trend was observed for AMI and ischemic stroke, while no consistent trend was detected for hemorrhagic stroke. The polynomial trend terms ($t$, ${t}^{2}$, ${t}^{3}$) indicated gradual temporal changes in hospitalization rates across the study period (*p* < 0.001 for AMI and *p* = 0.007 for ischemic stroke).

Regarding seasonal variation, hospitalizations for AMI showed a significant winter peak (IRR = 1.107, *p* < 0.001) and a reduction during summer (IRR = 0.946, *p* = 0.010), with fall serving as the reference category. A similar but weaker seasonal pattern was observed for ischemic stroke, with slightly higher admissions in winter (IRR = 1.046, *p =* 0.023). In contrast, hemorrhagic stroke did not show statistically significant seasonal changes (*p >* 0.05 across all seasons).

The annual harmonic terms (sin_year, cos_year) were not statistically significant, indicating that the seasonal variability was well captured by the calendar-season dummy variables rather than by continuous annual oscillation.

**Fig. 1:**
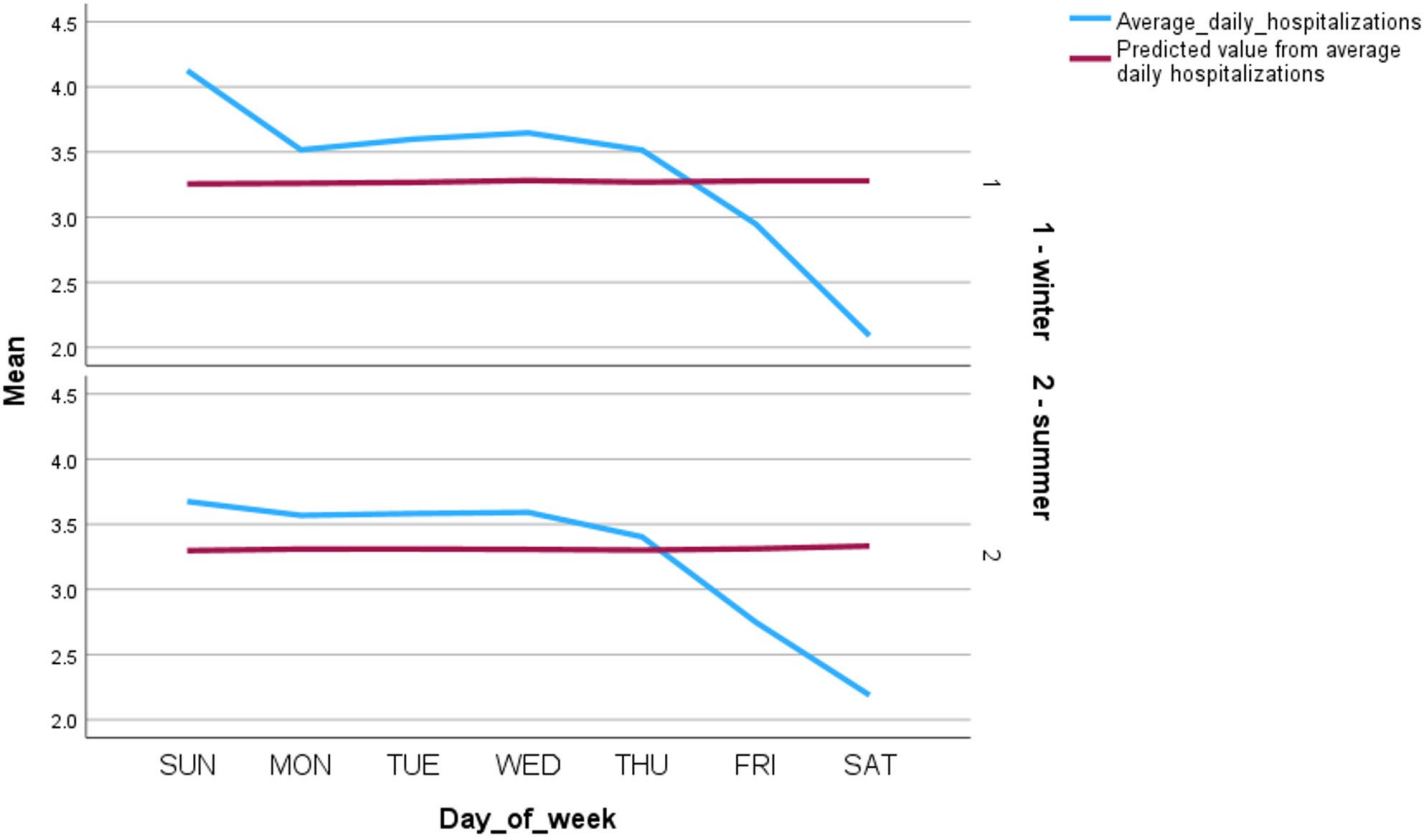
Mean daily hospitalizations for ischemic stroke at Rambam Medical Center, stratified by season (winter vs. summer) and day of the week

For the weekly cycle, no significant pattern was observed for AMI or hemorrhagic stroke, while a minor but significant effect was found for ischemic stroke (cos_week IRR = 0.978, *p* = 0.021), suggesting slightly lower admission rates toward the end of the week (Fig. 1).

Overall, the results show a clear winter dominance of cardiovascular and cerebrovascular hospitalizations in northern Israel, especially for myocardial infarction, consistent with previously observed cold-weather effects on acute cardiovascular events.

Table 3 summarizes the trend–harmonic analysis results for daily hospitalizations due to AMI, ischemic stroke, and hemorrhagic stroke at SMC from 2000 to 2018.

**Table 3:** Trend-harmonic analysis of daily CCD hospitalizations at SMC (2000–2018), by diagnosis

Variables	Acute Myocardial Infarction		Ischemic Stroke		Hemorrhagic Stroke	
	IRR^2^	*P*	IRR^1^	*P*	IRR^1^	*P*
Trend						
Intercept	2.550	< 0.001	5.916	< 0.001	1.394	< 0.001
* t*	1.001	< 0.001	0.999	< 0.001	1.000	0.730
* t* ^*2*^	1.000	< 0.001	1.000	0.008	1.000	0.879
* t* ^*3*^	1.000	< 0.001	1.000	0.038	1.000	0.914
Annual cycle						
Sin_year	0.977	0.339	1.009	0.490	1.001	0.975
Cos_year	0.984	0.494	1.010	0.450	0.972	0.226
Weekly cycle						
Sin_week	0.997	0.895	1.001	0.939	1.003	0.909
Cos_week	0.990	0.582	1.000	0.994	0.989	0.634
Seasonal cycle						
Winter	1.145	0.002	0.997	0.907	0.977	0.606
Spring	0.999	0.990	1.004	0.910	0.978	0.677
Summer	0.937	0.135	0.999	0.975	0.948	0.252
Fall (ref.)	1		1		1	

^1^Analysis was performed by Poisson regression

^2^Analysis was performed by Negative Binomial regression

*P* < 0.05

A significant long-term temporal trend was observed for AMI and ischemic stroke, whereas no significant trend was found for hemorrhagic stroke. The polynomial trend terms ($t$, ${t}^{2}$, ${t}^{3}$) indicated a gradual change in hospitalization rates over the study period (*p* < 0.001 for AMI and *p* < 0.05 for ischemic stroke).

In contrast to the northern region, the seasonal cycle in the southern (semi-arid) region showed a pronounced winter effect only for AMI (IRR = 1.145, *p* = 0.002), with no significant variation across other seasons. Ischemic and hemorrhagic stroke hospitalizations did not exhibit statistically significant seasonal differences (*p* > 0.05 for all seasons).

The annual harmonic components were not statistically significant for any diagnosis, suggesting that seasonal variation was mainly captured by the categorical season terms rather than continuous annual oscillations. Similarly, weekly periodicity was not observed for any of the three diagnostic groups, as none of the weekly sine–cosine parameters reached statistical significance.

Overall, in the semi-arid southern region, the findings show a significant increase in AMI hospitalizations during winter, with no similar seasonal pattern for stroke types. This contrasts with the northern Mediterranean region, where both AMI and ischemic stroke show mild winter peaks.

**Fig. 2:**
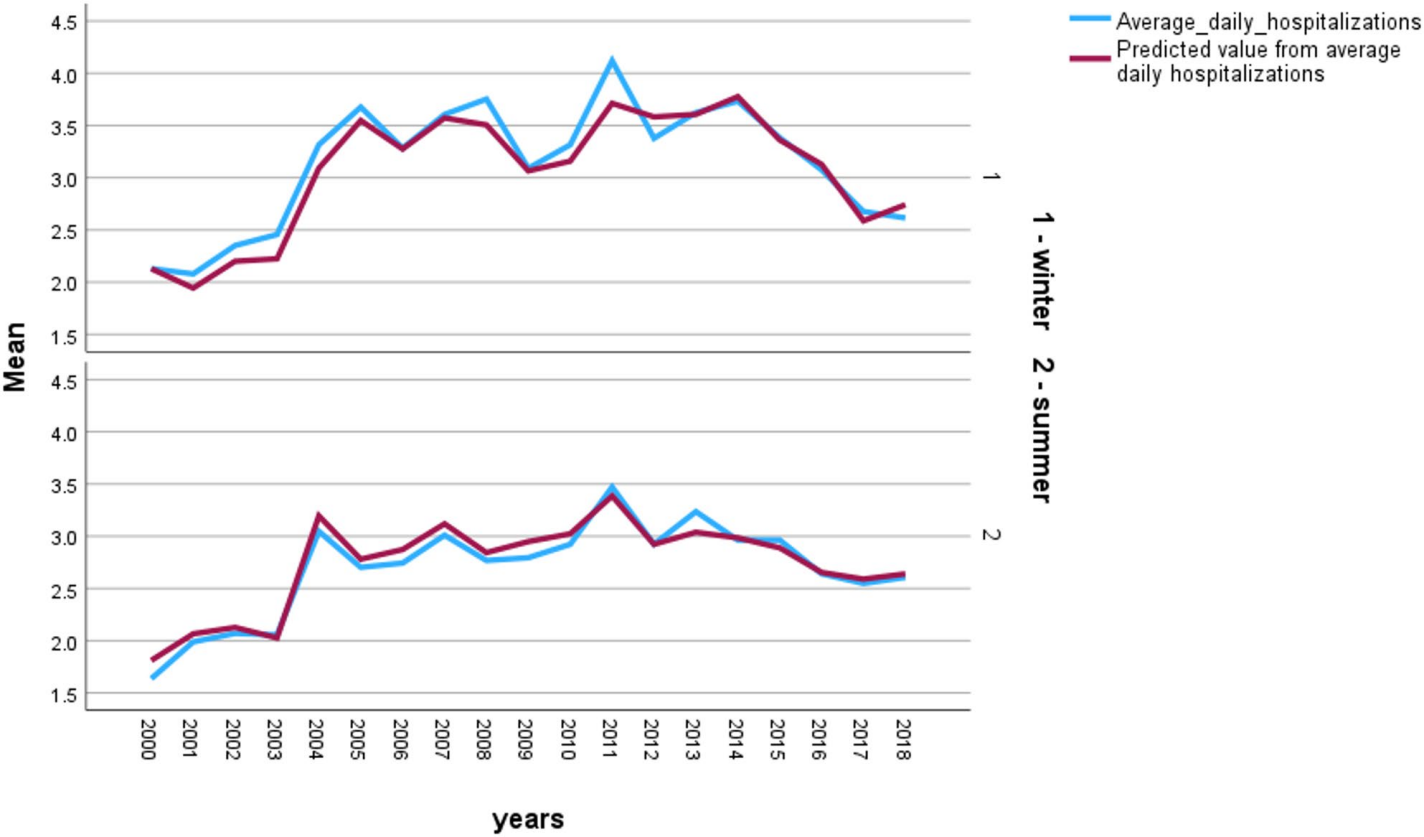
Mean daily hospitalizations for acute myocardial infarction at Rambam Medical Center, 2000–2018, stratified by season (winter vs. summer). Observed average daily counts are shown alongside model-predicted values

**Fig. 3:**
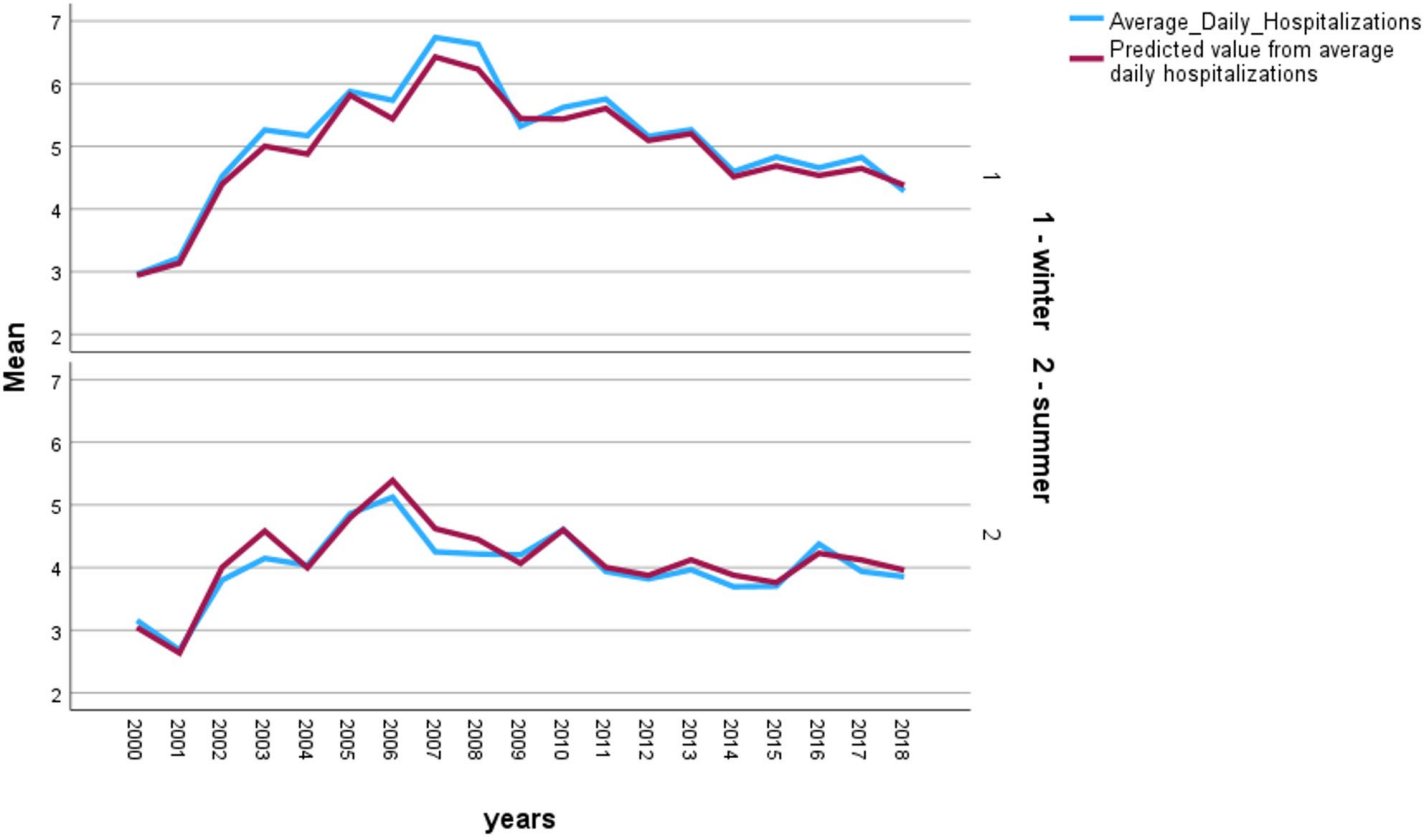
Mean daily hospitalizations for acute myocardial infarction at Soroka Medical Center, 2000–2018, stratified by season (winter vs. summer). Observed average daily counts are shown alongside model-predicted values

A clear seasonal pattern in AMI hospitalizations was observed at both medical centers. At RMC, the mean daily number of AMI hospitalizations peaked during winter (mean = 3.2) and reached its lowest level in summer (mean = 2.7) (Fig. 2). A similar pattern was observed at SMC, where the mean daily hospitalization rate was highest in winter (mean = 5.1) and lowest in summer (mean = 4.0) (Fig. 3). These findings are consistent with the trend-harmonic analysis, which demonstrated a significant winter increase in AMI admissions across both climatic regions.

## Discussion

This study analyzed almost two decades of hospitalizations due to cardiovascular and cerebrovascular diseases in two major hospitals located in climatically distinct regions of Israel. We identified a clear seasonal pattern in hospitalizations for acute myocardial infarction (AMI), with higher rates during winter and lower rates in summer, consistently observed in both northern (Rambam Medical Center) and southern (Soroka Medical Center) regions. A similar but weaker trend was noted for ischemic stroke, while hemorrhagic stroke did not show significant seasonality.

The winter increase in AMI admissions observed in our study is consistent with numerous previous reports from diverse geographic regions worldwide. Previous studies from the United States, Europe, and Asia have shown higher rates of AMI and cardiovascular mortality during winter months compared with summer [6, 13, 14]. Several physiological and environmental mechanisms have been proposed in previous studies to explain this pattern. Exposure to cold has been associated with increased sympathetic nervous activity, elevated blood pressure and heart rate, and peripheral vasoconstriction, all of which may promote plaque rupture and thrombosis [13, 14]. Cold weather has also been shown to raise blood viscosity, fibrinogen levels, and platelet aggregation, all of which can increase cardiovascular risk [15]. Additionally, respiratory infections, such as influenza, which are more common in winter, may trigger cardiovascular events [16].

Beyond external triggers, seasonal variation in AMI incidence may also reflect underlying differences in disease pathogenesis. Optical coherence tomography studies have shown a higher prevalence of plaque rupture in winter than in summer, whereas plaque erosion appears more common in warmer seasons. These findings support the notion that seasonality may influence the biological mechanisms underlying acute coronary syndromes, as well as their temporal distribution [17].

Interestingly, despite the generally warmer, semi-arid climate in southern Israel, a similar seasonal pattern of AMI hospitalizations was observed. This finding supports the hypothesis that not only low temperatures but also temperature variability, diurnal fluctuations, and sudden weather changes may contribute to the triggering of cardiac events [18, 19]. The difference in seasonal amplitude between regions could also reflect variations in population demographics, adaptation to weather, or socioeconomic factors.

Our findings align with previous studies conducted in Israel. Telman et al. [8] reported higher in-hospital mortality for ischemic stroke patients admitted during winter in northern Israel, while Yackerson et al. [9] demonstrated that atmospheric conditions in the southern desert region influence cardiac admissions. Libruder et al. [10] similarly identified a clear winter peak in stroke incidence across Israel over a 21-year period. By combining long-term data from two climatically distinct regions and applying harmonic trend modeling, our study offers a broader, integrated perspective on seasonal cardiovascular morbidity across different climate zones.

Recent literature has also emphasized that meteorological variability, beyond simple seasonal classification, may affect both the incidence and outcomes of ischemic stroke. Short-term changes in weather parameters, such as temperature, humidity, and precipitation, have been associated not only with stroke incidence but also with clinical outcomes, underscoring the importance of dynamic environmental conditions in cerebrovascular risk and prognosis [20]. These observations may partly explain the weaker yet still detectable seasonal trend in ischemic stroke identified in our analysis.

Seasonal patterns are also observed in other cardiovascular emergencies, including out-of-hospital cardiac arrest (OHCA). Although the incidence of OHCA varies by season, recent studies suggest that environmental exposures with seasonal variability have little effect on long-term survival. In a registry-based study examining the effects of particulate matter (PM₂.₅ and PM₁₀) and associated heavy metals on survival after OHCA, no independent association was found with either short-term or long-term survival after resuscitation. These findings suggest that seasonality may primarily influence the timing or triggering of acute cardiovascular events rather than long-term outcomes after their occurrence, underscoring the importance of distinguishing between factors that affect incidence and those that determine prognosis [21]. This distinction aligns with our findings, which primarily show seasonal variation in hospitalization rates rather than differences in in-hospital outcomes.

Seasonal variation is also observed in hospitalizations for acute decompensated heart failure (AHF). Large cohort studies, including Aronson (2021), have reported striking annual periodicity, with peaks in winter and the lowest rates in summer, independent of respiratory infections and other triggers. These findings suggest that, similar to AMI, winter months are associated with higher HF admissions, highlighting a broader pattern of seasonal cardiovascular vulnerability [22].

Understanding seasonal patterns in cardiovascular and cerebrovascular events has important implications for healthcare planning and prevention. Anticipating higher AMI admission rates in winter may support proactive allocation of hospital resources, staffing, and catheterization laboratory capacity. At the community level, maintaining medication adherence, promoting influenza vaccination, and increasing public awareness of cold-related cardiovascular risks may help reduce winter peaks in acute events [23].

This study has several limitations. As a retrospective, hospital-based ecological analysis, it cannot establish causal inferences. Detailed environmental exposure data, including temperature, humidity, air pollution, and atmospheric pressure, were unavailable, which represents a major limitation of the study; therefore, our analysis relied on regional proxies. Furthermore, individual-level cardiovascular risk factors, such as hypertension, diabetes mellitus, smoking status, and socioeconomic variables, were not available and may have contributed to the observed patterns.

Stroke subtypes were identified using ICD-9 codes, which may be subject to misclassification, particularly over a prolonged study period. This limitation is especially relevant for hemorrhagic stroke, for which no seasonal pattern was detected despite generally consistent coding protocols across hospitals. Nevertheless, the large sample size, extended 19-year observation period, and inclusion of two climatically distinct regions strengthen the robustness of our findings.

## Conclusion

In conclusion, we observed consistent winter peaks in hospitalizations due to acute myocardial infarction in both northern and southern Israel, demonstrating clear seasonal patterns in cardiovascular morbidity across different climatic regions. Given the study’s ecological design, the observed seasonality likely reflects a combination of environmental conditions, behavioral factors, healthcare-seeking patterns, and system-level influences rather than a direct causal effect of climate alone. Recognizing these patterns may support improved healthcare preparedness and resource allocation during the winter months.

## Data Availability

The datasets analyzed during the current study are not publicly available due to institutional restrictions but are available from the corresponding author on reasonable request.

## Abbreviations

AHF: Acute Heart Failure
AIC: Akaike Information Criterion
AMI: Acute Myocardial Infarction
BIC: Bayesian Information Criterion
CCD: Cardio-Cerebrovascular Disease
ICD: International Classification of Diseases
IRR: Incidence Rate Ratio
MI: Myocardial Infarction
OHCA: Out-of-Hospital Cardiac Arrest
RMC: Rambam Medical Center
SD: Standard Deviation
SMC: Soroka Medical Center
SPSS: Statistical Package for the Social Sciences
